# Down-Regulating the High Level of 17-Beta-Hydroxysteroid Dehydrogenase 13 Plays a Therapeutic Role for Non-Alcoholic Fatty Liver Disease

**DOI:** 10.3390/ijms23105544

**Published:** 2022-05-16

**Authors:** Meixi Wang, Jianrui Li, Hu Li, Biao Dong, Jing Jiang, Nannan Liu, Jiali Tan, Xuekai Wang, Lei Lei, Hongying Li, Han Sun, Mei Tang, Huiqiang Wang, Haiyan Yan, Yuhuan Li, Jiandong Jiang, Zonggen Peng

**Affiliations:** 1 CAMS Key Laboratory of Antiviral Drug Research, Institute of Medicinal Biotechnology, Chinese Academy of Medical Sciences & Peking Union Medical College, Beijing 100050, China; wangmx93@163.com (M.W.); lijianrui@imb.pumc.edu.cn (J.L.); lihu0112@163.com (H.L.); blizzarddon@vip.sina.com (B.D.); jiangjing3210@126.com (J.J.); lnnzzdxyxy@126.com (N.L.); tanjl2015@163.com (J.T.); wxk1002@126.com (X.W.); leilei010017@163.com (L.L.); lihongying018@163.com (H.L.); sh784696241@163.com (H.S.); tm1515409425@163.com (M.T.); hq_wangimb@163.com (H.W.); yan0495@163.com (H.Y.); yuhuanlibj@126.com (Y.L.); jiang.jdong@163.com (J.J.); 2 Beijing Key Laboratory of Antimicrobial Agents, Institute of Medicinal Biotechnology, Chinese Academy of Medical Sciences & Peking Union Medical College, Beijing 100050, China; 3 Key Laboratory of Biotechnology of Antibiotics, The National Health and Family Planning Commission (NHFPC), Institute of Medicinal Biotechnology, Chinese Academy of Medical Sciences & Peking Union Medical College, Beijing 100050, China

**Keywords:** 17-beta-hydroxysteroid dehydrogenase type 13, non-alcoholic fatty liver disease, steatosis, fibrogenesis, drug target

## Abstract

Non-alcoholic fatty liver disease (NAFLD) is the most common chronic liver disease worldwide, and there is no specific drug to treat it. Recent results showed that 17-beta-hydroxysteroid dehydrogenase type 13 (HSD17B13) is associated with liver diseases, but these conclusions are controversial. Here, we showed that HSD17B13 was more highly expressed in the livers of NAFLD patients, and high expression was induced in the livers of murine NAFLD models and cultural hepatocytes treated using various etiologies. The high HSD17B13 expression in the hepatocytes facilitated the progression of NAFLD by directly stabilizing the intracellular lipid drops and by indirectly activating hepatic stellate cells. When HSD17B13 was overexpressed in the liver, it aggravated liver steatosis and fibrosis in mice fed with a high-fat diet, while down-regulated the high expression of HSD17B13 by short hairpin RNAs produced a therapeutic effect in the NAFLD mice. We concluded that high HSD17B13 expression is a good target for the development of drugs to treat NAFLD.

## 1. Introduction

Non-alcoholic fatty liver disease (NAFLD) is a heterogeneous and comprehensive early liver disease, that ranges from noninflammatory isolated steatosis to non-alcoholic steatohepatitis (NASH) accompanied with severe liver fibrosis [1]. NAFLD frequently leads to advanced liver diseases, including cirrhosis and hepatocellular carcinoma (HCC), which cause liver failure leading to a high morbidity and mortality [2,3]. The NAFLD process is regulated by various genetic and environmental causative factors [4], but the underlying pathological mechanism for the development of NAFLD has yet to be further clarified [5,6]. To date, no specific drug has been approved for the treatment of NASH [7], though many candidates are in clinical trials [3,8], and some of them have shown promising results at the endpoints of their clinical trial [7,9].

Accumulative data indicated that 17-beta-hydroxysteroid dehydrogenase 13 (HSD17B13) is associated with the clinical outcomes of chronic liver diseases [10,11,12,13,14,15]. HSD17B13, which is located at chromosome 4q22.1, was first cloned in 2007 and was initially named short-chain dehydrogenases/reductase 9 (SCDR9) [16]. HSD17B13 belongs to the 17-β hydroxysteroid dehydrogenase superfamily (HSD17Bs), which contains fifteen members that respond to the oxidation and reduction of hormones, fatty acids, and bile acids [17,18]. Different from other members, HSD17B13 is mostly expressed in the liver and is mainly in charge of lipid metabolism, which is closely associated with the occurrence and development of chronic liver diseases [19,20,21]. In the early stages of liver diseases, HSD17B13 expression is enhanced [20]. Recently, clinical data from several clinical research centers have shown that the single nucleotide polymorphisms (SNPs) of HSD17B13, especially those of the rs72613567: TA allele, were associated with the progression and severity of NAFLD [11,22,23,24]. However, the medical literature on the genuine physiological function of HSD17B13 and on how it is associated with the outcomes of patients with NAFLD is very limited. High HSD17B13 expression led to liver steatosis in C57BL/6 mice [20], and subsequent studies demonstrated that liver X receptor alpha activation induced high HDS17B13 expression and thus led to the development of fatty liver disease [21]. However, HSD17B13 deficiency also resulted in liver steatosis [25]. These contradictory results suggest comprehensive roles of HSD17B13 in the development of NAFLD that remain to be demonstrated; whether HSD17B13 is a target for the development of drugs against NAFLD remains unclear.

In this study, we investigated the effects and mechanism of highly expressed HSD17B13 on the pathogenesis of NAFLD and validated whether HSD17B13 is a potential target for the development of drugs to treat NAFLD. 

## 2. Results

### 2.1. HSD17B13 Expression Is Increased in the Liver Tissues of Patients and Murine Models with NAFLD

**Figure 1:**
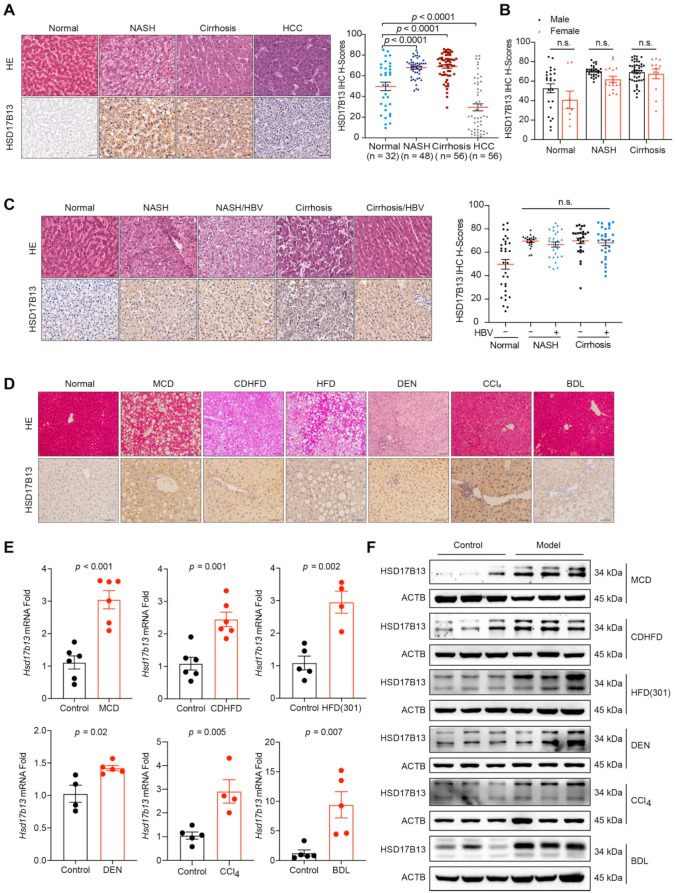
HSD17B13 expression is increased in the liver tissues of patients and murine models with NAFLD. (**A**–**C**) Representative H&E and immunohistochemical staining and the statistical summary in a human liver tissue array (**A**), the results were re-distinguished between man and woman (**B**), the results were re-distinguished with or without HBV infection (**C**). (**D**) C57BL/6 mice or SD rats were treated with pathogenic agents accordingly. Representative H&E and immunohistochemical staining of the murine livers. (**E**,**F**) mRNA (**E**) and protein (**F**) in the murine livers were measured by qRT-PCR and Western blotting, respectively. Results are expressed as mean ± SEM. (scale bars, 50 μm). NASH, non-alcoholic steatohepatitis; HCC, hepatocellular carcinoma; HBV, hepatitis B virus; MCD, methionine choline deficient diet; CDHFD, choline deficient and high-fat diet; HFD, high-fat diet; DEN, diethylnitrosamine; CCl_4_, carbon tetrachloride; BDL, bile-duct ligation.

To further validate the relationship between the HSD17B13 expression and NAFLD progression, we detected HSD17B13 expression in liver samples from patients with NAFLD using immunohistochemical analysis (Supplementary Table S1). Higher HSD17B13 expression was significantly observed in the livers with NASH or cirrhosis (Figure 1A), with the samples demonstrating IHC scores of 67.85 ± 1.37 in NASH and of 68.89 ± 1.71 in cirrhosis compared to the scores of 49.74 ± 4.13 found in normal livers (Figure 1A). However, HSD17B13 expression did not show a more significant increase in patients with cirrhosis (Figure 1A). Inversely, in the liver samples from patients with cancer, HSD17B13 expression was decreased, showing IHC scores of 29.52 ± 3.35 (Figure 1A), which were consistent with previous reports from patients with HCC [26]. Nevertheless, no significant gender differences were observed in HSD17B13 expression in the livers of NAFLD patients (Figure 1B).

Hepatitis B virus (HBV) infection is one of the major pathogenic factors causing chronic liver diseases [27,28]. We re-analyzed the data to distinguish the consequences from viral or non-viral infection. The results showed that HSD17B13 expression was no different between HBV infection and HBV un-infection in neither NASH nor in cirrhotic liver tissues (Figure 1C), though its expression was significantly up-regulated when compared to normal liver tissues (Figure 1C), suggesting that HSD17B13 expression is higher in the livers of patients with NAFLD, regardless of etiology.

To validate this assumption, we detected HSD17B13 expression in liver tissues from murine models of NAFLD induced by methionine-choline-deficient diet (MCD), choline-deficient and high-fat diet (CDHFD), high-fat diet (HFD), diethylnitrosamine (DEN), bile-duct ligation (BDL), and carbon tetrachloride (CCl_4_). Immunohistochemical analysis revealed that hepatic HSD17B13 expression in the livers of murine models with NAFLD (Supplementary Figure S1A,B) was markedly increased compared to in normal liver tissue samples (Figure 1D). Higher HSD17B13 expression confirmed via mRNA (Figure 1E) and protein levels (Figure 1F). These results hinted that the higher expression of HSD17B13 in livers with NAFLD is an outcome caused by etiological agents, regardless of the pathogenic factors.

### 2.2. Higher HSD17B13 Expression Is Mainly in Hepatocytes and Directly Induced by Etiological Agents

**Figure 2:**
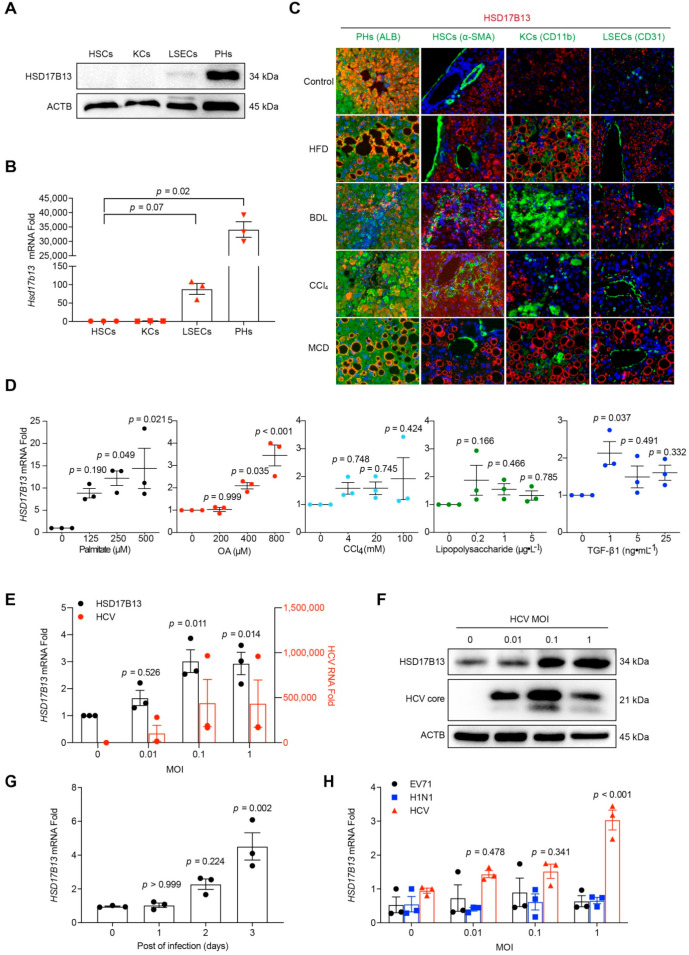
Higher HSD17B13 expression is mainly in hepatocytes and directly induced by etiological agents. (**A**,**B**) Protein (**A**) and mRNA (**B**) were detected in mouse primary cells. (**C**) Liver samples from NAFLD mice induced by HFD, BDL, CCl_4_, and MCD were processed with immunofluorescence co-staining for HSD17B13 with ALB (hepatocyte marker), CD31 (endothelial marker), α-SMA (activated HSC marker), or CD11b (macrophage marker). Slides were counterstained with DAPI (Scale bars, 50 μm). (**D**) L02 cells were treated by palmitate, OA, CCl_4_ lipopolysaccharide, or TGF-β1 for 24 h, mRNA was measured by qRT-PCR. (**E**,**F**) Huh7.5 cells were infected with HCV, RNAs were measured by qRT-PCR (**E**), and proteins were measured by Western blotting (**F**) at 72 h. (**G**) Huh7.5 cells were infected by HCV (MOI = 0.1) and mRNA was measured by qRT-PCR at designated days post-infection. (**H**) mRNA in respective host cells infected by HCV, H1N1 and EV71 was measured by qRT-PCR. Results are expressed as mean ± SEM. HSCs, hepatic stellate cells; KCs, Kupffer cells; LSECs, liver sinusoidal endothelial cells; PHs, hepatic parenchymal cells; HFD, high-fat diet; BDL, bile-duct ligation; CCl_4_, carbon tetrachloride; MCD, methionine-choline-deficient diet; OA, oleic acid; TGF-β1, transforming growth factor-beta 1; MOI, multiplicity of infection; HCV, hepatitis C virus; H1N1, influenza A virus; EV71, enterovirus type 71.

HSD17B13 is especially expressed in the livers shown in gene databases [29,30], and recent single-cell sequencing data have shown that HSD17B13 expression is restricted to hepatocytes [31]. We further verified its distribution in the liver according to protein levels (Figure 2A). In cells that were isolated from the liver tissues of C57BL/6 mice, HSD17B13 was mainly expressed in hepatic parenchymal cells (PHs), while it was dramatically lower in other cell types in the liver, including liver sinusoidal endothelial cells (LSECs), Kupffer cells (KCs), and hepatic stellate cells (HSCs) (Figure 2A). The results were confirmed at the mRNA level (Figure 2B).

To detect whether the higher expression of HSD17B13 remained in the PHs after treating the causative agents, we detected the distribution of HSD17B13 in mouse livers after treatment of causative agents. The results showed that the higher expression of HSD17B13 was, identically, mainly in the PHs of mouse NAFLD models that were induced by HFD, BDL, CCl_4_, or MCD, showing with the co-localization of HSD17B13 with PHs (albumin, ALB) (Figure 2C). While the expression of HSD17B13 was still low in other cell types in the liver, including in LSECs (platelet/endothelial cell adhesion molecule 1, CD31), HSCs (alpha-smooth muscle actin, α-SMA), and KCs (integrin alpha M, CD11b) (Figure 2C). These results suggested that the induced higher expression of HSD17B13 is also restricted to PHs.

We then further validated whether the causative agents directly induce the higher expression of HSD17B13. Hepatocytes were treated with palmitate, oleic acid (OA), lipopolysaccharide, transforming growth factor-beta 1 (TGF-β1), and CCl_4_, respectively. The up-regulated expression of HSD17B13 was significantly induced by treatment with palmitate or OA in L02, while the other agents were only slightly up-regulated at certain concentrations but without significance (Figure 2D). The outcomes were similar in the treated Huh7 (Supplementary Figure S2A) and HepG2 cells (Supplementary Figure S2B). The results indicated that the pathogenic factors directly induced the higher expression of HSD17B13.

Notably, hepatitis C virus (HCV) infection is one of the leading etiological agents resulting in progressive liver fibrosis [32,33]. Therefore, we analyzed the expression of HSD17B13 after HCV infection in Huh7.5 cells, a hepatocyte cell line that strongly supports HCV infection and replication [34,35]. After three days of HCV infection, HSD17B13 expression was increased and paralleled with HCV viral loads (Figure 2E,F). Additionally, it also increased gradually over the course of three days during HCV infection (Figure 2G). The high expression of HSD17B13 was consistent with the results from clinical samples infected with HBV (Figure 1B), and this could be because HBV and HCV belong to the family of hepatotropic viruses. In contrast, infections of non-hepatotropic viruses, such as enterovirus type 71 (EV71) and influenza A virus (H1N1), did not change HSD17B13 expression in the corresponding hosted cultural cells (Figure 2H), suggesting that HSD17B13 might be up-regulated by specific causative agents associated with chronic liver diseases.

### 2.3. HSD17B13 Promotes the Accumulation of Intracellular Lipid Droplets and Causes Hepatocyte Injury

**Figure 3:**
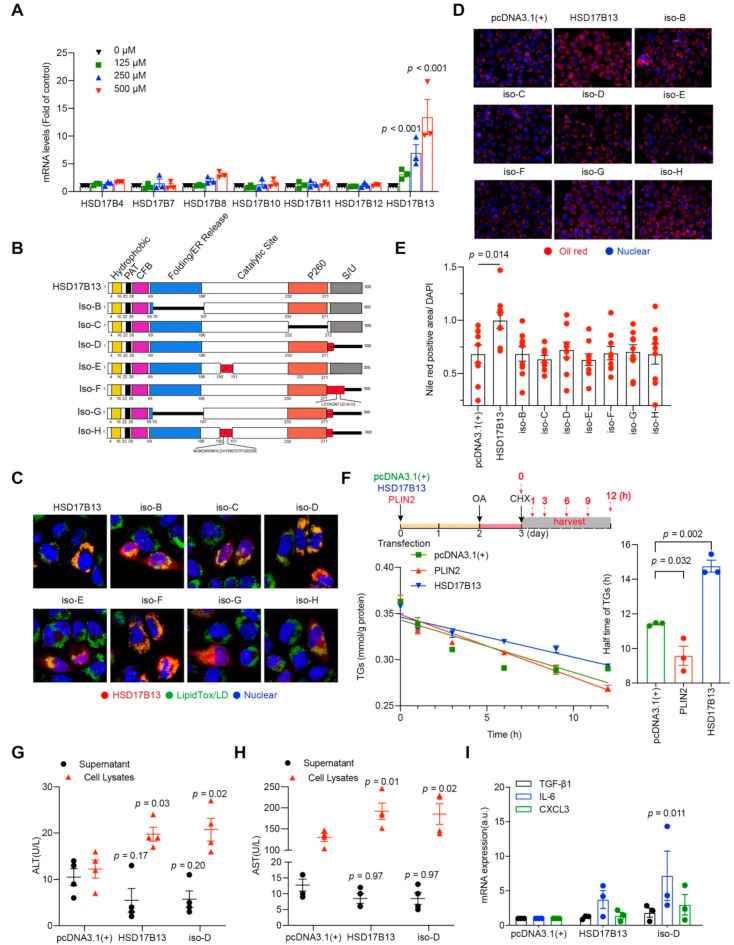
HSD17B13 promotes the accumulation of intracellular lipid droplets and causes hepatocyte injury. (**A**) mRNAs in palmitate-treated cells were measured by qRT-PCR. (**B**) Schematic of full-length or truncated transcripts of HSD17B13. (**C**) Representative confocal images of L02 cells transfected with His-tagged HSD17B13 transcripts (red) and LDs (green) after being induced by OA (400 μM) for 24 h. Nuclei were labeled with DAPI (blue). (**D**,**E**) Representative immunofluorescence staining images for LDs (red) (**D**) and relative intracellular Nile red positive areas were quantified (**E**) in L02 cells transfected with HSD17B13 transcripts after OA (400 μM) challenge for 24 h, the slides were counterstained with DAPI. (**F**) Schematic of the experimental design for investigating the effect of HSD17B13 on intracellular TGs, the contents of TGs were detected and the half-life of TGs was calculated. (**G**,**H**) Levels of ALT (**G**) and AST (**H**) in supernatants and cell lysates of Huh7 cells transfected with the plasmids of HSD17B13 for 24 h. (**I**) mRNA of proinflammatory markers in L02 cells transfected with plasmids of HSD17B13 for 24 h. Results are expressed as mean ± SEM. OA, oleic acid; LDs, lipid droplets; TGs, triglycerides; PLIN2, perilipin 2; ALT, alanine transaminase; AST, aspartate transaminase; TGF-β1, transforming growth factor-beta 1; IL-6, interleukin 6; CXCL3, C-X-C motif chemokine ligand 3.

In the HSD17B superfamily, HSD17B4, HSD17B7, HSD17B8, HSD17B10, HSD17B11, and HSD17B12 have been reported be related to cholesterol and fatty acid metabolisms [17] and thus may have the same effect as HSD17B13 does. To verify the specificity of HSD17B13 expression in liver diseases, we detected their expression in a palmitate-induced cell model. Only HSD17B13 expression showed a significant up-regulation (Figure 3A). The result suggested that the HSD17B13 of the superfamily responds to the inducing agent specifically.

HSD17B13 contains eight transcripts that range from A to H (Figure 3B) [11]. Among them, HSD17B13 transcript A encodes the full-length protein of HSD17B13, which was reported to be a lipid droplet (LD)-associated protein [19]. However, whether the other HSD17B13 transcripts are also related to LDs remains to be clarified. After the transfection of the highly expressed plasmids with the His-tag at the C-terminal amino acid of the protein into the L02 cells, HSD17B13 was also co-localized with LDs (Figure 3C), hinting that the His-tag does not influence the intracellular localization of HSD17B13. Similarly, all of the other transcripts were co-localized with LDs (Figure 3C), suggesting that HSD17B13 may be related to LDs metabolism, regardless of transcripts.

We then investigated whether the high expression of the eight transcripts directly affects intracellular fat contents. In L02 cells incubated with a high concentration of OA, HSD17B13 increased the contents of LDs, while the other transcripts did not increase (Figure 3D,E), suggesting that HSD17B13, the full-length protein, might be the main causative factor for the early occurrence of NAFLD, especially the liver steatosis.

To determine the mechanism underlying the effect of HSD17B13 on LDs, HepG2 cells were transfected with the highly expressed HSD17B13 plasmids and were then treated with OA after two days. The intracellular triglyceride (TG) levels were analyzed after treatment with cycloheximide (CHX) to block protein synthesis (Figure 3F). The half-life of the TGs was slightly decreased by the high expression of perilipin 2 (PLIN2) (Figure 3F), which was reported to facilitate TGs degradation [36,37] and was significantly increased by the overexpression of HSD17B13 (Figure 3F), suggesting that HSD17B13 stabilizes intracellular TGs contents and thus accumulates intracellular LDs.

A recent large-scale genome-wide association study (GWAS) identified that HSD17B13 and HSD17B13 transcript D (iso-D) were associated with the plasma levels of alanine transaminase (ALT) and aspartate transaminase (AST) [11,38]. These increased transaminase levels occurred concomitantly with metabolic syndrome risk factors and were related to liver injury and fibrosis [39]. To analyze whether HSD17B13 directly influences transaminases levels, Huh7 cells were transfected with the highly expressed HSD17B13 plasmids, and the transaminase levels were detected. The over-expressed HSD17B13 showed decreased transaminase levels in culture supernatants and significantly increased transaminases levels in cell lysates (Figure 3G,H). This suggests that the high level of HSD17B13 found in the hepatocytes might be a direct cause of hepatocyte injury. 

In patients with NAFLD, liver injury is accompanied by inflammation [40,41]. In L02 cells that have been transfected with the highly expressed HSD17B13 plasmids, the mRNA levels of interleukin 6 (IL-6), C-X-C motif chemokine ligand 3 (CXCL3) and TGF-β1 were increased (Figure 3I), and the IL-6 levels demonstrated a significant increase caused by transfection with iso-D. These results suggested that cell injury caused by increased HSD17B13 levels in hepatocytes might occur through the IL-6 dependent inflammatory pathway.

### 2.4. HSD17B13 in Hepatocytes Indirectly Promotes the Activation of HSCs

**Figure 4:**
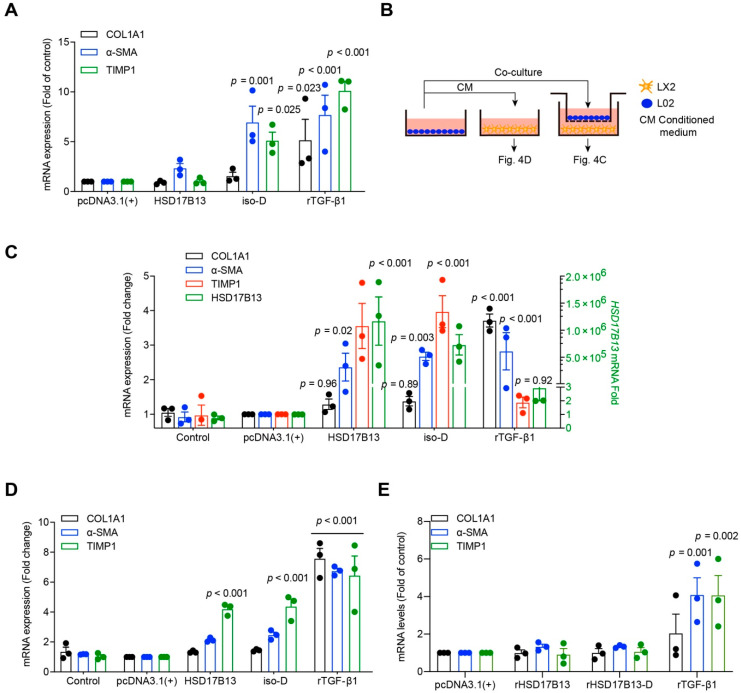
HSD17B13 in hepatocytes indirectly promotes the activation of HSCs. (**A**) mRNA was quantitated with qRT-PCR in LX2 cells transfected with plasmids for 24 h. (**B**–**D**) Schematic flow chart of Transwell insert mono- and co-culture models (**B**), mRNA was quantitated in LX2 cells from the co-cultured with L02 cells transfected with plasmid (**C**) or cultured with CM from plasmid-transfected L02 cells (**D**). (**E**) mRNA levels in LX2 cells cultured with recombinant protein (2 ng/mL) for 24 h. Results are expressed as mean ± SEM. COL1A1, collagen type I alpha 1 chain; α-SMA, alpha-smooth muscle actin; TIMP1, TIMP metallopeptidase inhibitor 1; rTGF-β1, transforming growth factor-beta 1 recombinant protein.

Persistent liver injury, especially persistent inflammation, activates HSCs [42]. The activated HSCs play a pivotal profibrogenic role in liver fibrosis [42,43]. Notably, we observed that when HSD17B13 was highly expressed, it was mainly distributed around the activated HSCs (Figure 2C). Although HSD17B13 expression is extremely low in HSCs (Figure 2A,B), we tested whether high HSD17B13 expression in HSCs will impact HSC activation. After being transfected with high plasmid expression, the LX2 (a human hepatic stellate cell line) cells were not activated by HSD17B13 (Figure 4A).

HSD17B13 expression is very low in HSCs but high in PHs in both normal and NAFLD models (Figure 2A–C). To further investigate the role of HSD17B13 during HSC activation, we used a co-culture system with hepatocytes and HSCs. The LX2 cells were co-cultured with L02 cells that were transfected with the highly expressed HSD17B13 plasmids, and LX2 cells were cultured with the cultural supernatants (CM) from the transfected L02 cells (Figure 4B). The results showed that the LX2 cells were significantly activated by means of co-culturing with the transfected L02 cells, showing collagen type I alpha 1 chain (*COL1A1*), *α-SMA*, and TIMP metallopeptidase inhibitor 1 (*TIMP1*) with higher markers representing activated HSCs (Figure 4C) that were partially activated by the CM from the transfected L02 cells with the HSD17B13 plasmid (Figure 4D). However, normal cell culture media plus exogenous HSD17B13 could not dramatically induce the HSC activation as much as TGF-β1 did (Figure 4E), further ruling out a role from HSD17B13 itself in the supernatant. These results suggest that the activation of HSCs may be indirectly induced by potential factors secreted by hepatocytes that have been transfected with plasmids with high HSD17B13 expression.

### 2.5. Overexpression of HSD17B13 in the Liver Aggravates Liver Steatosis and Fibrosis in HFD-Fed Mice

**Figure 5:**
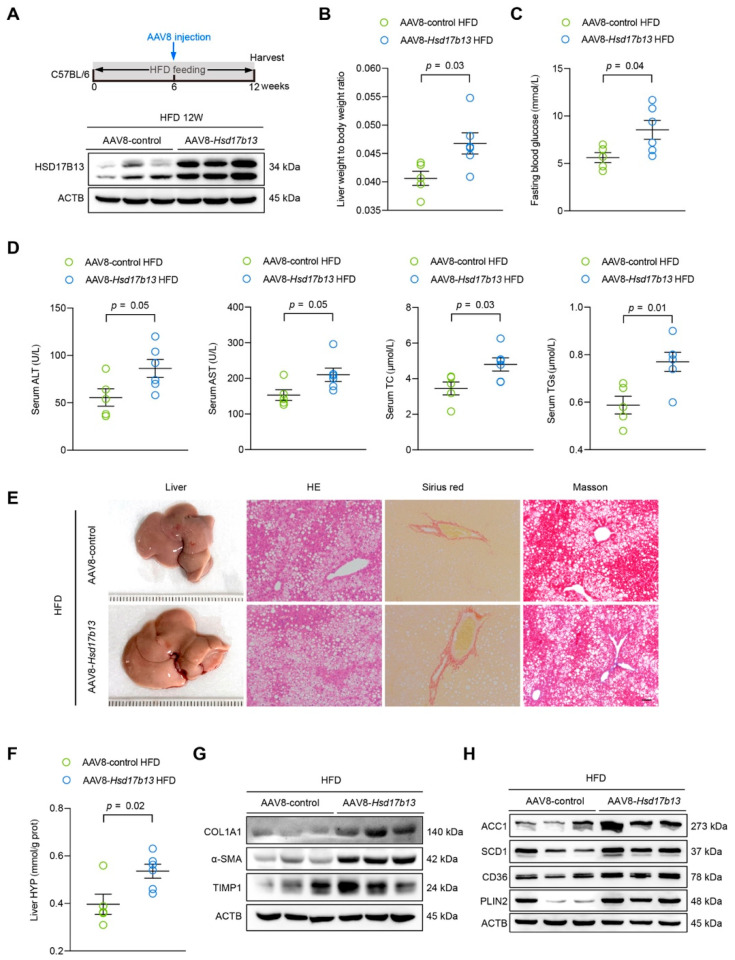
Overexpression of HSD17B13 in the liver aggravates liver steatosis and fibrosis in HFD-fed mice. (**A**) Schematic of the experimental design of AAV8-*Hsd17b13* treatment in an HFD-induced mouse model, and HSD17B13 proteins in the mouse livers were detected with Western blotting at 12 weeks. (**B**) Liver weight to body weight ratio. (**C**) Fasting blood glucose concentration. (**D**) Serum concentrations of ALT, AST, TC, and TGs. (**E**) Representative liver histology of H&E, Sirius red, and Masson staining (Scale bars, 20 μm). (**F**) Liver HYP. (**G**) Representative Western blots of related fibrosis protein in the mouse livers. (**H**) Representative Western blots of protein related to lipid accumulation in the mouse livers. Results are expressed as mean ± SEM. HFD, high fat diet; AAV8, adeno-associated virus 8; ALT, alanine transaminase; AST, aspartate transaminase; TC, total cholesterol; TGs, triglycerides; HYP, hydroxyproline; COL1A1, collagen type I alpha 1 chain; α-SMA, alpha-smooth muscle actin; TIMP1, tissue inhibitor of metalloproteinase 1; ACC1, acetyl-Coenzyme A carboxylase alpha; SCD1, stearoyl-Coenzyme A desaturase 1; CD36, cluster of differentiation 36; PLIN2, perilipin 2.

Previous results showed that the overexpression of HSD17B13 in the liver in normal mice for four days induced markedly increased neutral lipid accumulation [20]. To further investigate the pathological role of the high HSD17B13 expression in the liver, we used an injection of adeno-associated virus 8 (AAV8) carried with plasmids of HSD17B13 to specifically overexpress HSD17B13 (AAV8-*Hsd17b13*) in the livers of HFD-fed mice for six weeks (Figure 5A). As expected, AAV8-*Hsd17b13* strongly increased HSD17B13 expression in the liver compared to the AAV8-control (Figure 5A). Higher HSD17B13 expression significantly increased the liver coefficient (Figure 5B) as well as the fasting blood glucose levels (Figure 5C) and significantly induced higher levels of ALT, AST, total cholesterol (TC), and TGs in serum (Figure 5D). HFD-induced liver hepatic steatosis and fibrosis were also significantly aggravated by higher HSD17B13 expression inducted by the AAV8-*Hsd17b13* injection, something that was visible with histopathology (Figure 5E), paralleled with the increased liver hydroxyproline (HYP) expression (Figure 5F) as well as the expression of related fibrosis proteins (Figure 5G). Furthermore, the overexpression of HSD17B13 markedly increased the levels of the proteins related to lipid accumulation (Figure 5H), such as acetyl-coenzyme A carboxylase alpha (ACC1) and stearoyl-Coenzyme A desaturase 1 (SCD1), which are related to lipogenesis; cluster of differentiation 36 (CD36), which is related to lipid uptake; and lipid coat protein PLIN2, which agreed with our results in vitro (Figure 3).

**Figure 6:**
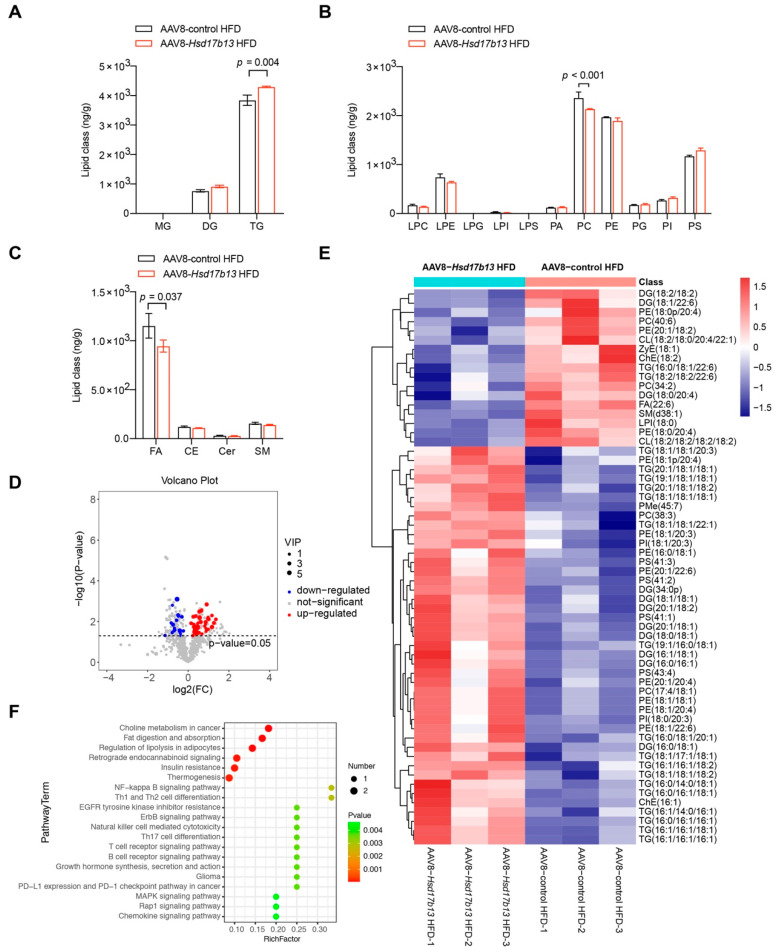
Overexpression of HSD17B13 in the liver alters lipid metabolisms in HFD-fed mice. (**A**–**C**) Alterations of hepatic lipid classes in the livers of HFD-fed mice injected with AAV8-*Hsd17b13*. (**D**) Volcano plot of differentially expressed lipid metabolisms. (**E**) Heatmaps of the expression of significant lipid class. (**F**) KEGG pathway analysis. HFD, high fat diet; MG, monoglyceride; DG, diacylglycerol; TG, triacylglycerol; LPC, lysophosphatidylcholine; LPE, lysophosphatidyl ethanolamine; LPG, lysophosphatidylglycerol; LPI, lysophosphatidyl inositol; LPS, lysophosphatidylserine; PA, phosphatidic acid; PC, phosphatidylcholine; PE, phosphatidylethanolamine; PG, phosphatidylglycerol; PI, phosphatidylinositol; PS, phosphatidylserine; FA, free fatty acid; CE, cholesteryl ester; Cer, ceramide; SM, sphingomyeline.

To validate the potential pathogenic mechanism of HSD17B13, we performed lipid metabolomics using ultra-performance liquid chromatography/mass spectrometry (LC/MS). After injection with AAV8-*Hsd17b13*, the total triacylglycerol (TG) content (Figure 6A) was significantly increased, while the total phosphatidylcholine (PC) (Figure 6B) and free fatty acid (FA) contents (Figure 6C) were significantly decreased, and no alterations were observed in the other lipid types (Figure 6A–C). The multi-dimensional analysis and single-dimensional analysis results showed that 60 lipid metabolisms were differentially expressed (VIP > 1, q < 0.05) in the mice that had been injected with AAV8-*Hsd17b13* (Figure 6D), with 17 of down-regulation and 43 of up-regulation. This difference is mainly due to the subtle lipid ingredients (Figure 6E), suggesting that the changes in the lipid metabolisms were caused by HSD17B13. The Kyoto Encyclopedia of Genes and Genomes (KEGG) pathway analysis results showed that the overexpression of HSD17B13 mainly influenced lipid metabolism- and inflammation-related pathways, including fat digestion and absorption, the regulation of lipolysis in adipocytes, the NF-κB signaling pathway and MAPK signaling pathway (Figure 6F), demonstrating agreement with our results regarding high HSD17B13 expression in hepatocytes (Figure 3).

These results suggested that the higher levels of HSD17B13 in the liver plays a pathogenic role in the occurrence and progression of NAFLD via lipid metabolism- and inflammation-related pathways, and therefore, down-regulating the high level of HSD17B13 might be a strategy to prevent the occurrence of NAFLD and to attenuate the progressive NAFLD.

### 2.6. Down-Regulation of HSD17B13 Plays a Therapeutic Role in NAFLD Mice

**Figure 7:**
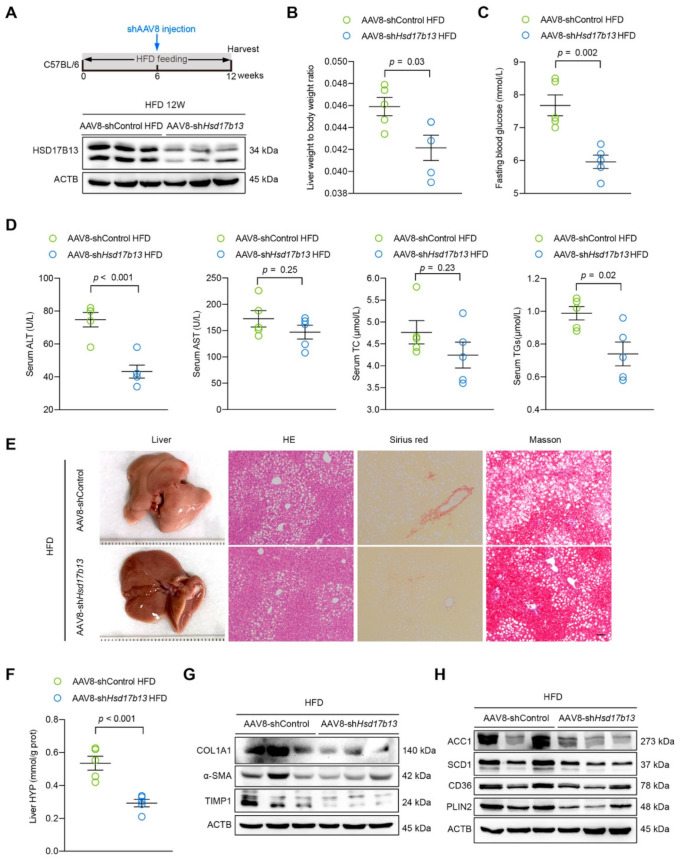
Down-regulation of HSD17B13 plays a therapeutic role in NAFLD mice. (**A**) Schematic of the experimental design of AAV8-sh*Hsd17b13* treatment in an HFD-induced mouse model, and HSD17B13 protein level in the mouse livers was detected with Western blotting at 12 weeks. (**B**) Liver weight to body weight ratio. (**C**) Fasting blood glucose concentration. (**D**) Serum concentrations of ALT, AST, TC, and TGs. (**E**) Representative liver histology of H&E, Sirius red and Masson staining (Scale bars, 20 μm). (**F**) Liver HYP. (**G**) Representative Western blots of related fibrosis protein in the mouse livers. (**H**) Representative Western blots of protein related to lipid accumulation in the mouse livers. Results are expressed as mean ± SEM. HFD, high fat diet; AAV8, adeno-associated virus 8; ALT, alanine transaminase; AST, aspartate transaminase; TC, total cholesterol; TGs, triglycerides; HYP, hydroxyproline; COL1A1, collagen type I alpha 1 chain; α-SMA, alpha-smooth muscle actin; TIMP1, tissue inhibitor of metalloproteinase 1; ACC1, acetyl-Coenzyme A carboxylase alpha; SCD1, stearoyl-Coenzyme A desaturase 1; CD36, cluster of differentiation 36; PLIN2, perilipin 2.

To validate our hypotheses, we used short hairpin RNAs (shRNA) of HSD17B13 delivered by AAV8 (AAV8-sh*Hsd17b13*) to specifically knock down HSD17B13 in the livers of HFD-fed mice for six weeks (Figure 7A). The down-expression of HSD17B13 (Figure 7A) via AAV8-sh*Hsd17b13* injection reduced the high liver coefficient (Figure 7B) and fasting blood glucose (Figure 7C) in the HFD-fed mice. In parallel, HSD17B13 knockdown significantly decreased the serum levels of ALT and TGs compared to those in the HFD-fed control mice (Figure 7D). Notably, lower HSD17B13 expression induced by the injection of AAV8-sh*Hsd17b13* improved the characteristics of NAFLD induced by HFD remarkably, including hepatocyte steatosis and fibrosis (Figure 7E,F), and the outcomes were associated with the decreased HSC activation (Figure 7G) and the expression of proteins associated with lipid metabolisms (Figure 7H). 

**Figure 8:**
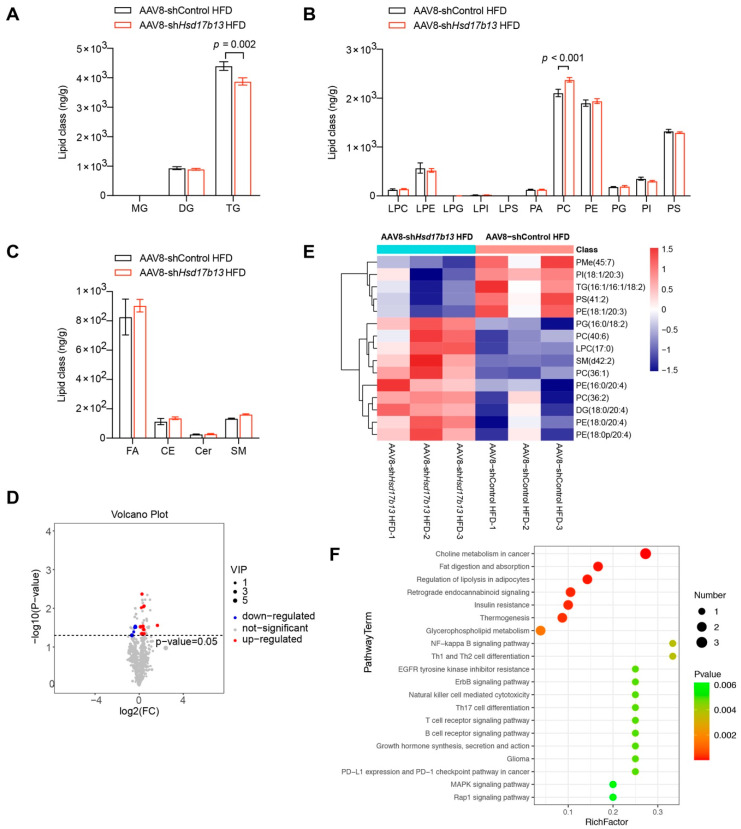
Down-regulation of HSD17B13 in the liver improves lipid metabolisms in HFD-fed mice. (**A**–**C**) Alterations of hepatic lipid classes in the liver of HFD-fed mice injected with AAV8-sh*Hsd17b13*. (**D**) Volcano plot of differential lipid metabolisms. (**E**) Heatmaps of the expression of significant lipid class. (**F**) KEGG pathway. HFD, high fat diet; MG, monoglyceride; DG, diacylglycerol; TG, triacylglycerol; LPC, lysophosphatidylcholine; LPE, lysophosphatidyl ethanolamine; LPG, lysophosphatidylglycerol; LPI, lysophosphatidyl inositol; LPS, lysophosphatidylserine; PA, phosphatidic acid; PC, phosphatidylcholine; PE, phosphatidylethanolamine; PG, phosphatidylglycerol; PI, phosphatidylinositol; PS, phosphatidylserine; FA, free fatty acid; CE, cholesteryl ester; Cer, ceramide; SM, sphingomyeline.

The lipid metabolomics results showed that in the livers of the mice injected with AAV8-sh*Hsd17b13*, the total TG content was significantly decreased (Figure 8A), while the total PC was significantly increased (Figure 8B), without altering other lipid types (Figure 8A–C). Fifteen lipid metabolisms, with five down-regulated and ten up-regulated, were differentially expressed (VIP > 1, q < 0.05) in the mice injected with AAV8-sh*Hsd17b13* (Figure 8D). Notably, PC (36:2), PC (40:6) and PC (36:1) were elevated (Figure 8E), while TG (16:1/16:1/18:1) (Figure 8E) was reduced in the AAV8-sh*Hsd17b13* mice, showing the opposite of what was observed after AAV8-*Hsd17b13* injection (Figure 6E). The KEGG pathway analysis showed that the involved pathways in the mice injected with AAV8-sh*Hsd17b13* were the same as those in the mice injected with AAV8-*Hsd17b13* Figure 6F and Figure 8F). Thus, our findings suggested that the down-regulation of the high level of HSD17B13 produced a therapeutic effect on NAFLD via improving the abnormal liver lipid metabolism and inflammation; therefore, it might be a good strategy for the treatment of NAFLD, as HSD17B13 expression was normally higher in NAFLD that had been induced by various etiological agents (Figure 1).

## 3. Discussion

In this work, we showed that HSD17B13 expression was up-regulated in the livers of patients with NAFLD and increased in the livers of multiple murine NAFLD models and hepatocytes treated with etiological agents. When HSD17B13 was more highly impressed, it, both directly and indirectly, played pathogenic roles in the occurrence and development of NAFLD in vitro and in vivo, and thus down-regulating the high expression of HSD17B13 produced a therapeutic effect in NAFLD.

HSD17B13 is an LD-associated protein [44] and is mainly expressed in the liver [19]; its biochemical function is related to cholesterol and fatty acid metabolism [17]. We further verified that HSD17B13 was restricted to PHs in both normal livers and etiological agent-treated livers (Figure 2C). The higher expression of HSD17B13 was directly induced by various factors, including chemical, immunological, fatty, and drug agents, and infections caused by hepatotropic viruses (Figure 1, Figure 2D–G and Supplementary Figure S2). These results suggested that higher HSD17B13 expression might be a key factor related to the occurrence and progression of NAFLD. Therefore, we paid more attention to the pathogenic roles of the higher expression of HSD17B13 in NAFLD. However, how the increased expression of HSD17B13 is induced by a common or different mechanism requires further investigation, although reports have shown that it is related with LXRs or SREBP-1c [21]. We found that all of the HSD17B13 transcripts were co-localized with LDs (Figure 3C) due to their sharing of the N-terminal 1-35 amino acid sequences, which are sufficient for HSD17B13 to localize to LDs [19,45]. Our results further suggested that HSD17B13 played an important role in LD metabolism, which agreed with the role of HSD17Bs [17]. Using this clue, we found that HSD17B13 prevented the cells from degrading intracellular TGs, significantly prolonging the half-life of TGs in hepatocytes (Figure 3D), though the detailed mechanism of how HSD17B13 stabilizes LDs needs to be further demonstrated. As such, the intracellularly accumulated TGs may lead to the occurrence of liver steatosis due to the fact that long-term over-accumulated TGs are one of the pathogenetic markers of early chronic liver diseases [46,47]. Furthermore, long-term redundant LDs commonly accompany liver injury and inflammation [47,48] and hence facilitate the progression of NAFLD [49,50]. We found that the high expression of HSD17B13 induced LD accumulation and directly caused liver injury as well as an increase in intracellular ALT and AST accompanied by the higher expression of inflammatory factors (Figure 3E–G). However, the internal connecting link between the high expression of HSD17B13 and inflammation remains to be clarified.

What attracts our attention is that a large amount of HSD17B13 was distributed around the activated HSCs in the livers of the mice treated with etiological agents (Figure 2C), suggesting that HSD17B13 may be associated with the activation of HSCs in the mouse liver. However, exogenous human HSD17B13 showed slight involvement in HSC activation in vitro (Figure 4E), while the HSCs were indirectly activated significantly when co-cultured with hepatocytes with high HSD17B13 expression or by the cultural supernatants of hepatocytes with high HSD17B13 expression (Figure 4C,D). Therefore, it is more possible that the highly expressed HSD17B13 indirectly activated the HSCs, which would have further aggravated the progress of early NAFLD. The activated HSCs may also be related to the roles of the inflammatory factors that were increased by hepatocyte injury due to the highly expressed HSD17B13. Certainly, the associated factors that were induced by HSD17B13 were different from those that were induced by TGF-β1 because the biomarkers of the HSCs that were activated by co-culture or by means of supernatants were differently induced (Figure 4C,D). However, we could not exclude the direct activation of HSCs by the highly expressed HSD17B13, especially in vivo. In addition, HSD17B13 stabilized intracellular TGs and thus might cause unbalance of LDs in the liver, which might hinder autophagy and lipolysis processes, and sequentially worsen liver steatosis to NASH [47].

We demonstrated that the high expression of HSD17B13 facilitated the occurrence and development of early liver diseases (Figure 5). The pathogenic mechanism was associated with enhancing the protein expressions of lipid synthesis, uptake, and lipid coat protein, which are related to the accumulation of LDs (Figure 5H). A recent study described how the genetic variants of HSD17B13 decreased the risk of NAFLD by influencing the metabolism of hepatic phospholipids [51]. Our lipid metabolomics study further indicated that the amounts of TG were significantly elevated in the mouse livers with over-expressed HSD17B13, while the PC contents were significantly decreased (Figure 6), suggesting that TG and PC play an important role in HSD17B13-related NAFLD. Furthermore, high HSD17B13 expression also promoted HSC activation, increasing the biomarkers of activated HSCs (Figure 5G). These results are in agreement with previously reported clinical results, further suggesting that HSD17B13 mainly influences the outcomes of early liver diseases [11,22,51]; the mechanism is consistent with our in vitro results (Figure 3). Furthermore, the down-regulation of the more highly expressed HSD17B13 by shRNA attenuated liver diseases (Figure 7). This therapeutic effect might result from the down-regulated expressions of the LD-associated proteins and the enhanced activation of HSCs, which also validates the possible pathogenic mechanism of more highly expressed HSD17B13. However, HSD17B13 deficiency also showed an exacerbation of NAFLD in the mouse models [25,31]. As such, we presumed that a homeostatic level of HSD17B13 might be very important in NAFLD, a conclusion that requires further investigation, especially when considering whether there are similar or different mechanisms that cause NAFLD.

In summary, our findings showed that HSD17B13 is commonly more highly expressed in the livers of patients and mice with NAFLD that was induced by various etiological agents. Higher HSD17B13 expression facilitates the accumulation of LDs in the liver and promotes the activation of HSCs, thus increasing the occurrence and development of NAFLD. Down-regulating HSD17B13 by shRNA in HFD-fed mice attenuates NAFLD via improving the abnormal liver lipid metabolism and inflammation. Therefore, down-regulating the high expression of HSD17B13 in the liver is a promising strategy for the development of drugs to treat NAFLD.

## 4. Materials and Methods

### 4.1. Animal Experiments 

The 6- to 8-week-old male C57BL/6 mice (25–27 g) and male Sprague Dawley (SD) rats (150–170 g) with SPF grade were from Beijing HuaFuKang Biological Technology Co. Ltd. (Beijing, China). All of the animals were kept in a standard 12 h light–dark cycle under SPF conditions and were allowed free access to water and food. For the HFD-induced liver disease model, mice were fed with HFD (D09100301 [52], Research Diets, New Brunswick, NJ, USA; H10060, Beijing HFK Bioscience, Beijing, China) or corresponding control diet for 16 weeks. For the CDHFD- [53] induced liver disease model, mice were fed with CDHFD (M10560, MolDiets, Beijing, China) or a control diet for 16 weeks. For the model of DEN- [53] induced liver disease, SD rats were given 100 ppm of DEN (N0756, Sigma, Darmstadt, Germany) to drink for 6 weeks. For the CCl_4_- [53] induced liver disease model, the mice were injected with corn oil alone (vehicle control mice) or with CCl_4_ (Tianjin Fuchen Chemical Reagent Factory, Tianjin, China; 1 mL/kg, in corn oil at a ratio of 1:4, i.p., twice a week for 6 weeks). The mice were sacrificed 24 h after the last injection of CCl_4_. For the BDL- [54] induced liver disease model, the common bile duct was doubly ligated with 4-0 silk and transected between the ligated sites. Sham-operated mice were operated on similarly as the control, except the bile duct was not ligated or transected. Liver tissues and serum samples were collected on the fifth day after the operation. For the MCD (1%)-HFD diet- [55] induced liver disease model, the mice were fed with MCD (1%)-HFD (M10462S, MolDiets, Beijing, China) diet or control diet for 6 weeks. The liver tissues and serum samples were collected and stored for subsequent analysis. All animal experiments were conducted following the National Guidelines for Housing and Care of Laboratory Animals. The study protocols were approved by the Institutional Animal Care and Use Committee of the Institute of Medicinal Biotechnology & Chinese Academy of Medical Sciences (SYXK(Jing)2017-0023).

### 4.2. Cell Culture 

Human HSCs, LX2 cells [56], were kindly provided by Dr. Hong-wei He (Peking Union Medical College, Beijing, China). LX2 cells were cultured in DMEM GlutaMAX-I (10566016, Gibco, New York, NY, USA) with 10% FBS (16000-044, Gibco, New York, NY, USA) and 1% penicillin–streptomycin (PS, 15140122, Gibco, New York, NY, USA). A549 cells were cultured in F-12K (21127030, Gibco, New York, NY, USA) supplemented with 10% FBS and 1% PS. All cells were incubated under 5% CO_2_ under a water-saturated atmosphere at 37 °C. 

### 4.3. Cell Treatment

Oleate (OA, O1008, Sigma-Aldrich, Darmstadt, Germany) and palmitate (P0500, Sigma-Aldrich, Darmstadt, Germany) were solubilized in a phosphate-buffered saline (PBS, 20012050, Gibco, New York, NY, USA) solution by heating it to 55 °C and 65 °C, respectively. Solubilized fatty acids were conjugated with 10% fatty acid free-bovine serum albumin (BSA, B2064, Sigma-Aldrich, Darmstadt, Germany) in culture medium to generate a fatty acid stock solution. TGF-β1 (100-21-10, Peprotech, Suzhou, Jiangsu, China) was dissolved according to the manufacturer’s instructions. CCl_4_ was added to the full medium and dissolved completely after being positioned in a CO_2_ incubator for 48 h to form a saturated solution. For the model of the hepatocytes treated with causative agents, L02, Huh7, and HepG2 cells were seeded in 6-well plates, respectively. After adherence, the cells were incubated with palmitate, OA, CCl_4_, lipopolysaccharide, or TGF-β1 at different concentrations for another 24 h, respectively. For the proinflammatory factors assay, L02 cells were seeded in 6-well plates and transfected with indicated plasmids in the figure legends. After 6 h of transfection, the cells were incubated with fresh medium for 24 h, and then they were collected for further analysis.

### 4.4. Virus Infection

Huh7.5 cells were infected with genotype 2a JFH-1/J6 HCV virus [57] for the indicated time. HCT-8 cells were infected with EV71 (ATCC, VR-1432) virus for 1 h and fresh medium was replenished for further culture for 72 h [58]. A549 cells were infected with the influenza A (H1N1, strain A/FM1/1/47) virus for 2 h and replaced with F-12K supplemented with 2% FBS and 1% PS to culture for 24 h [59,60]. Intracellular RNAs and proteins were extracted and detected. 

### 4.5. Isolation of Primary Hepatocytes

Mouse primary hepatic parenchymal cells (6-0514-A, CHI Scientific, Jiangyin, Jiangsu, China), liver sinusoidal endothelial cells (3-4659-A, CHI Scientific, Jiangyin, Jiangsu, China), Kupffer cells (3-4722-A, CHI Scientific, Jiangyin, Jiangsu, China), and HSCs (6-0521-A, CHI Scientific, Jiangyin, Jiangsu, China) were isolated, respectively, from C57BL/6 mouse livers using isolation kits according to the manufacturer’s instructions.

### 4.6. Nile Red Staining Assay

L02 cells were seeded in 6-well plates and transfected with plasmids of HSD17B13 transcripts. After 24 h culture, the cells were re-seeded in specific climbing slides in 24-well plates and then induced by adding OA stock solution into culture media with a final concentration of 400 μM for 24 h. The cells were fixed with 3% paraformaldehyde and permeabilized with 0.5% Triton X-100. Nile Red solution at 10 μM concentration was added to incubate for 10 min. The images were acquired using Zeiss Axio observer X-cite series 120 microscope (Zeiss, Jena, Germany) at the magnification of 63×. The quantification of lipid droplets was performed using Image J.

### 4.7. Confocal Assays

L02 cells were transfected with the indicated plasmids with His-tag. After 6 h of transfection, the cells were exposed to OA at a final concentration of 400 μM for 24 h. The cells were stained with anti-His antibody followed by the corresponding fluorophore-conjugated secondary antibody and then stained with lipidTox (H34475, Invitrogen, New York, NY, USA) for 1 h at 37 °C. Images were acquired using a confocal microscope (LSM710, Zeiss, Jena, Germany).

### 4.8. TG Turnover Study

TG was tested as per the previous description [61]. Briefly, HepG2 cells were transfected with pcDNA3.1(+), PLIN2 and HSD17B13 plasmids, respectively. The media were changed after 48 h of transfection, and the cells were incubated with OA (400 µM). After 24 h, the medium was replaced with regular growth media mixed with cycloheximide (CHX, 100 µg/mL; C7698, Sigma-Aldrich, Darmstadt, Germany). Intracellular TGs were extracted from the cells at designated times in the figure legends and quantified using detection kits (E1013, Applygen, Beijing, China). The protein contents in the cells were quantified using the BCA protein assay kit (23225, Thermo Scientific, New York, NY, USA) and the half-time of the TGs was calculated by the fitting curve.

### 4.9. Co-Culture of Hepatocytes and HSCs

A Transwell co-culture system was used in this study [54,62]. LX2 cells were pre-cultured in 12-well plates for 24 h with 0% FBS, and L02 cells (with or without HSD17B13 transfection) were seeded in Transwell inserts (3401, Costar, Kennebunk ME, USA) that were subsequently loaded into the HSC-containing wells. The base of the insert has a membrane with 0.4 μm pores, which are large enough to permit free exchange of secreted proteins between cell types. LX2 and L02 cells were collected and analyzed after 48 h of co-culture. The transforming growth factor β1 recombinant protein (rTGFβ1) (2 ng/mL) was used as a positive control.

### 4.10. Intracellular Transaminase Analyses

Huh7 cells were seeded in 6-well plates and transfected with HSD17B13 plasmids. The media were changed after 6 h of transfection and were replenished with fresh media to culture for 24 h. The supernatant of the Huh7 cells was collected separately. The cells were harvested by 160 μL M-PER lysis buffer (78501, Thermo Scientific, New York, NY, USA) containing 1 mmol/L protease inhibitor cocktail (C0001, Targetmol, Shanghai, China), and then the supernatants were collected after 16,000× *g* centrifugation for 10 min. ALT and AST were measured using the Hitachi 7100 Clinical Analyzer (Hitachi, Japan).

### 4.11. Western Blot Analyses and Antibodies

The proteins were extracted from the mouse liver tissues using T-PER lysis buffer (78510, Thermo Scientific, New York, NY, USA) with a protease inhibitor cocktail. Proteins were extracted from the cells using M-PER lysis buffer with a protease inhibitor cocktail. All of the proteins were quantified using a BCA protein assay kit. Protein samples were separated on suitable concentration SDS-PAGE gels and transferred to PVDF membranes (IPVH00010; Millipore, Billerica, MA, USA). The membranes were blocked with 5% skim milk in TBST, and then incubated with the indicated primary antibodies overnight at 4 °C. After being washed with TBST, the membranes were incubated with secondary horseradish peroxidase (HRP)-conjugated antibodies for 1 h at room temperature. Protein expression signals were detected using a ChemiDoc MP Imaging System (Bio-Rad, Hercules, CA, USA). Beta-actin (ACTB) was used as the equal loading control. All of the antibodies used in this study are listed in Supplementary Table S2.

### 4.12. Plasmid Construction

The full-length, deletion, and point mutant fragments of HSD17B13 were generated using standard PCR methods and then subcloned into the corresponding pcDNA3.1(+) vectors. Other truncated transcripts of HSD17B13 were determined using Taihe Biotechnology (Beijing, China). The pcDNA3.1(+)-TGF-β1-His plasmid and pcDNA3.1(+)-TGF-β1 plasmid were constructed as previously described [63]. The pcDNA3.1(+)-PLIN2 plasmid was constructed using standard PCR methods and then subcloned into the corresponding pcDNA3.1(+) vectors. All plasmids were confirmed by sequencing. The primers used for plasmid construction are listed in Supplementary Table S3.

### 4.13. Expression and Purification of Recombinant Proteins

pcDNA3.1(+)-HSD17B13-His plasmid or pcDNA3.1(+)-TGF-β1-His plasmid was transfected into HEK293T cells. The culture medium and the cells were collected after 72 h. Recombinant HSD17B13 and TGF-β1 were purified using Ni Sepharose High-Performance following the manufacturer’s instruction. Finally, the recombinant protein was concentrated with a 10k-MW cutoff filter (UFC5010BK, Millipore Sigma, St. Louis, MO, USA). The purified recombinant protein was stored at −80 °C.

### 4.14. Quantitative Real-Time PCR Analyses

Total RNA was extracted using the Total RNA kit (QIAGEN, Dusseldorf, Germany). The RNA concentration was determined using a NanoDrop ND-1000 spectrophotometer (NanoDrop Inc., Wilmington, DE, USA). RNAs were detected using the HiScript II One Step qRT-PCR SYBR Green Kit (Vazyme, Nanjing, Jiangsu, China). The amplification conditions were: 50 °C for 15 min; 95 °C for 30 s; 40 cycles of 95 °C for 10 s, 60 °C for 34 s. The mRNA expression levels of the target genes were normalized to *GAPDH* using the 2^−ΔΔCt^ method. The detailed primer pairs used in this study are shown in Supplementary Table S4.

### 4.15. Quantification of HCV RNA

Using an RNeasy Mini Kit (QIAGEN, Dusseldorf, Germany), the total RNA was extracted from the Huh 7.5 cells and was detected using the AgPath-ID One-Step RT-PCR Kit (Applied Biosystems, Foster City, CA, USA). qRT-PCR analysis was performed using a previously described protocol [63]. *GAPDH* was served as the internal control for the quantification procedure. The primer and probe sequences are provided in Supplementary Table S4.

### 4.16. Histopathologic Analysis

Briefly, H&E staining was performed on paraffin-embedded liver tissues to visualize the lipid accumulation pattern and inflammatory status. Liver fibrosis was assessed via Sirius red and Masson’s trichrome staining. Histopathological images were acquired with a light microscope (Olympus, Tokyo, Japan). 

### 4.17. Immunofluorescence Staining

For dual immunofluorescence staining, liver sections were fixed with ice-cold methanol and co-stained with HSD17B13 (rabbit polyclonal; Thermo Fisher, New York, NY, USA) or other corresponding antibodies for detection by the appropriate secondary antibodies labeled with either Alexa Fluor 488 or Alexa Fluor 546, according to the manufacturer’s instructions. Nuclei were stained with DAPI. 

### 4.18. Fasting Blood Glucose Test

To determine fasting blood glucose concentration, blood from the mouse tails was examined using a blood glucose meter (Perform, Roche, Basel, Switzerland) following 12 h of fasting at the end of the experiments.

### 4.19. Liver Function and Biochemical Test

The levels of several biochemical markers were measured to assess liver function and liver injury in the mice. The measured biochemical markers in the mouse serum, including ALT, AST, TC and TGs, were measured using the Hitachi 7100 Clinical Analyzer (Hitachi, Japan). The HYP contents in the liver tissue were performed using commercial kits (A030-3 for HYP, Nanjing JianCheng Bioengineering Institute, Nanjing, Jiangsu, China), according to the manufacturer’s instructions.

### 4.20. Adenovirus Vector Construction and Infection

Adenoviruses were generated by Genechem CO., Ltd. (Shanghai, China). Specifically, the entire coding regions of mouse *Hsd17b13* were cloned into the pAAV-ApoE/hAATp-MCS vector separately. To knock down *Hsd17b13* expression in the mouse livers, mouse short hairpin RNAs (shRNAs) specific to the *Hsd17b13* gene were separately cloned into the pAAV-ApoE/hAATp-EGFP-MIR155 (MCS) vector. The mature shRNA sequences were: 5′-GCGTCATCATCTACTCCTACC-3′. The recombinant adenoviruses were plaque-purified and titered to 1 × 10^12^ plaque-forming units (PFU) per milliliter. AAV8-control and AAV8-shControl (nonsense sequence: 5′-TTCTCCGAACGTGTCACGT-3′) were used as controls for overexpression or knockdown, respectively. For in vivo experiments, the 6- to 8-week-old male mice were fed with HFD (D09100310, Research Diets, New Brunswick, NJ, USA) for 12 weeks and injected with 100 μL of virus containing 4 × 10^11^ AAV8 vector genomes or 2 × 10^11^ shAAV8 vector genomes in the tail vein at the sixth week [64], respectively.

### 4.21. Lipid Metabolomics Analysis

Three representative mice in each group were selected to perform lipid metabolomics analysis using ultra-performance liquid chromatography-mass spectrometry (LC/MS) by Shanghai LuMing Biological Technology Co. Ltd. (Shanghai, China). Briefly, samples were separated using a Nexera UPLC (Shimadzu, Kyoto, Japan) and analyzed by mass spectrometry with Q Exactive (Thermo Scientific). Heated electrospray ionization (HESI) was used for detection in the positive and negative ion modes, respectively. Differentially expressed lipid metabolisms were identified using the DESeq R package functions estimateSizeFactors and nbinomTest. A *p* value of <0.05 and fold change of >2 or fold change of <0.5 were set as the threshold values for significantly differential expression. The KEGG pathway enrichment analysis of the differential expressed lipid metabolisms were respectively performed using R based on the hypergeometric distribution.

### 4.22. Immunohistochemical Staining

Immunohistochemistry analyses were performed using paraffin-embedded sections of tissue samples. Human liver tissue samples were obtained from human liver tissue microarray slide (DC-liv00009, Avilabio, Xian, China). HSD17B13 in the liver tissue samples was detected using a rabbit anti-HSD17B13 antibody (PA5-56063 for human liver tissue, 1:300 dilution, Thermo Fisher; NBP3-04837 for mouse liver tissue, 1:100 dilution, NOVUS). The tissues were incubated with primary antibodies overnight at 4 °C and then with an HRP-conjugated secondary antibody. Immunohistochemical staining of liver tissue microarray slide was visualized using the Densito quant module of Quant Center 2.1. The intensity of staining was determined by the following rules: 0 for negative; 1 for weak staining; 2 for moderate staining; and 3 for strong staining. The final score was quantified by the H-Score (H-SCORE = ∑(PI × I) = (percentage of cells of weak intensity × 1) + (percentage of cells of moderate intensity × 2) + percentage of cells of strong intensity × 3).

### 4.23. Statistics

All data were analyzed using appropriate statistical analysis methods with GraphPad (Prism V.8) and showed as mean ± SEM. The Student’s t-test was used to analyze the differences between two groups, while one-way ANOVA was applied for multiple comparisons. A *p* value of less than 0.05 was considered statistically significant. 

## Data Availability

The data associated with this paper are available upon request to the corresponding author.

## Supplementary Materials

The following supporting information can be downloaded at: https://www.mdpi.com/article/10.3390/ijms23105544/s1.
