# Supplementary material for: Down-Regulating the High Level of 17-Beta-Hydroxysteroid Dehydrogenase 13 Plays a Therapeutic Role for Non-Alcoholic Fatty Liver Disease
Source: Int J Mol Sci. 2022 May 16;23(10):5544. doi: 10.3390/ijms23105544 (PMC9146021; doi:10.3390/ijms23105544)
Supplement: Supplementary file 1 [file ijms-23-05544-s001.zip › ijms-1688666-supplementary.pdf]

Article

Down-Regulating the High Level of 17-Beta-Hydroxysteroid Dehydrogenase 13 Plays a Therapeutic Role for Non-Alcoholic Fatty Liver Disease

Meixi Wang [1], Jianrui Li [1,2], Hu Li [1,3], Biao Dong [1,3], Jing Jiang [1], Nannan Liu [1], Jiali Tan [1], Xuekai Wang [1], Lei Lei [1], Hongying Li [1], Han Sun [1], Mei Tang [1], Huiqiang Wang [1], Haiyan Yan [1,2], Yuhuan Li [1,2,3], Jiandong Jiang [1,2,3] and Zonggen Peng [1,2,3,*]

[1] CAMS Key Laboratory of Antiviral Drug Research, Institute of Medicinal Biotechnology, Chinese Academy of Medical Sciences & Peking Union Medical College, Beijing 100050, China

[2] Beijing Key Laboratory of Antimicrobial Agents, Institute of Medicinal Biotechnology, Chinese Academy of Medical Sciences & Peking Union Medical College, Beijing 100050, China

[3] Key Laboratory of Biotechnology of Antibiotics, The National Health and Family Planning Commission (NHFPC), Institute of Medicinal Biotechnology, Chinese Academy of Medical Sciences & Peking Union Medical College, Beijing 100050, China

SUPPLEMENTARY FIGURES

Supplementary Figure S1

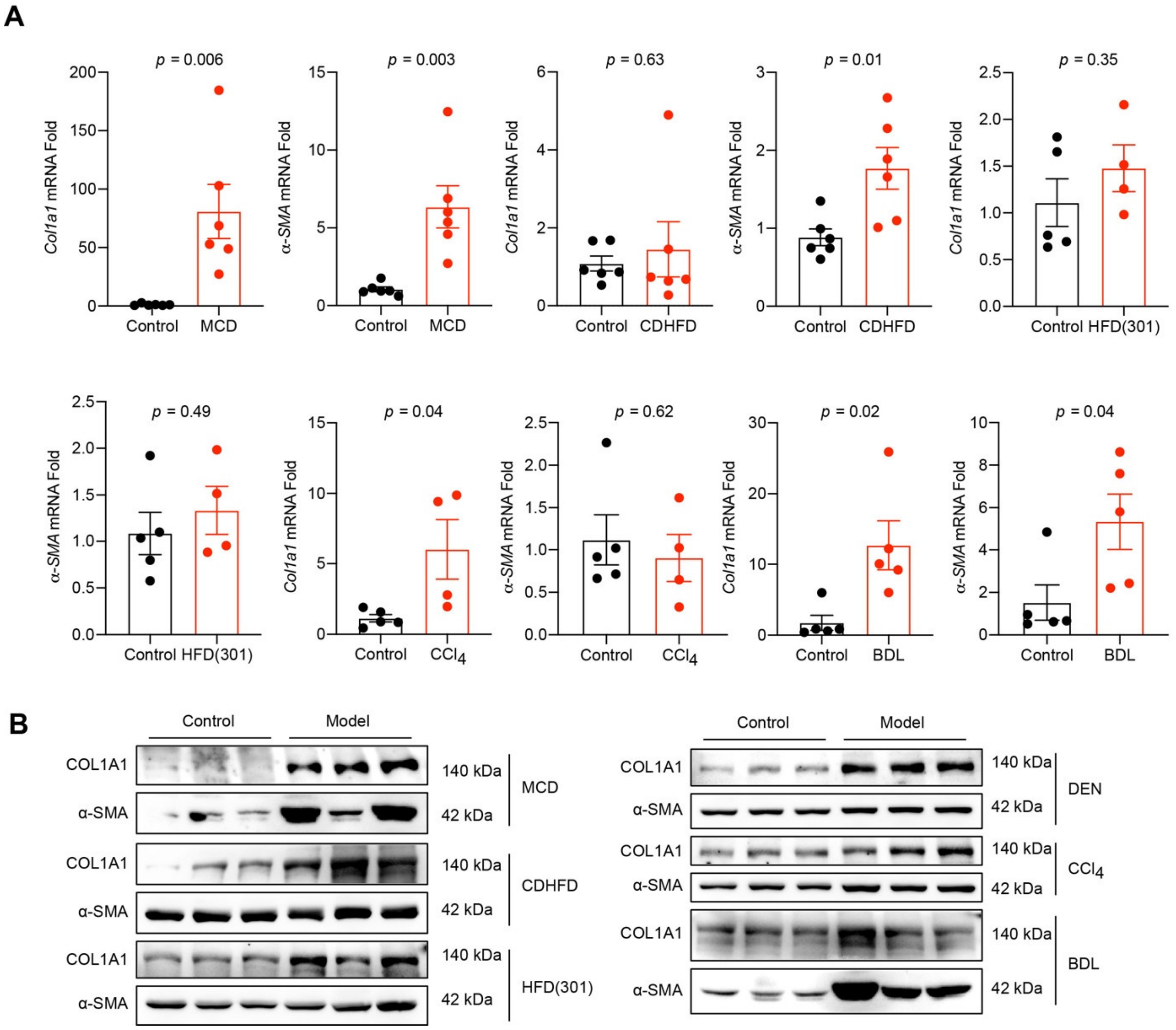

Supplementary Figure S1. Fibrosis-related factors are highly expressed in the livers of murine with NAFLD. mRNA (A) and protein (B) levels in the livers of murine models with NAFLD were measured by qRT-PCR and Western blotting, respectively. The equal loading control (ACTB) of protein was shown in **Figure 1E**. Results are expressed as mean ± SEM. CCl_4, carbon tetrachloride; MCD, methionine choline deficient diet; CDHFD, choline deficient and high-fat diet; HFD, high-fat diet; DEN, diethylnitrosamine; BDL, bile-duct ligation; Col1a1, collagen type I alpha 1 chain; α-SMA, alpha-smooth muscle actin.

Supplementary Figure S2

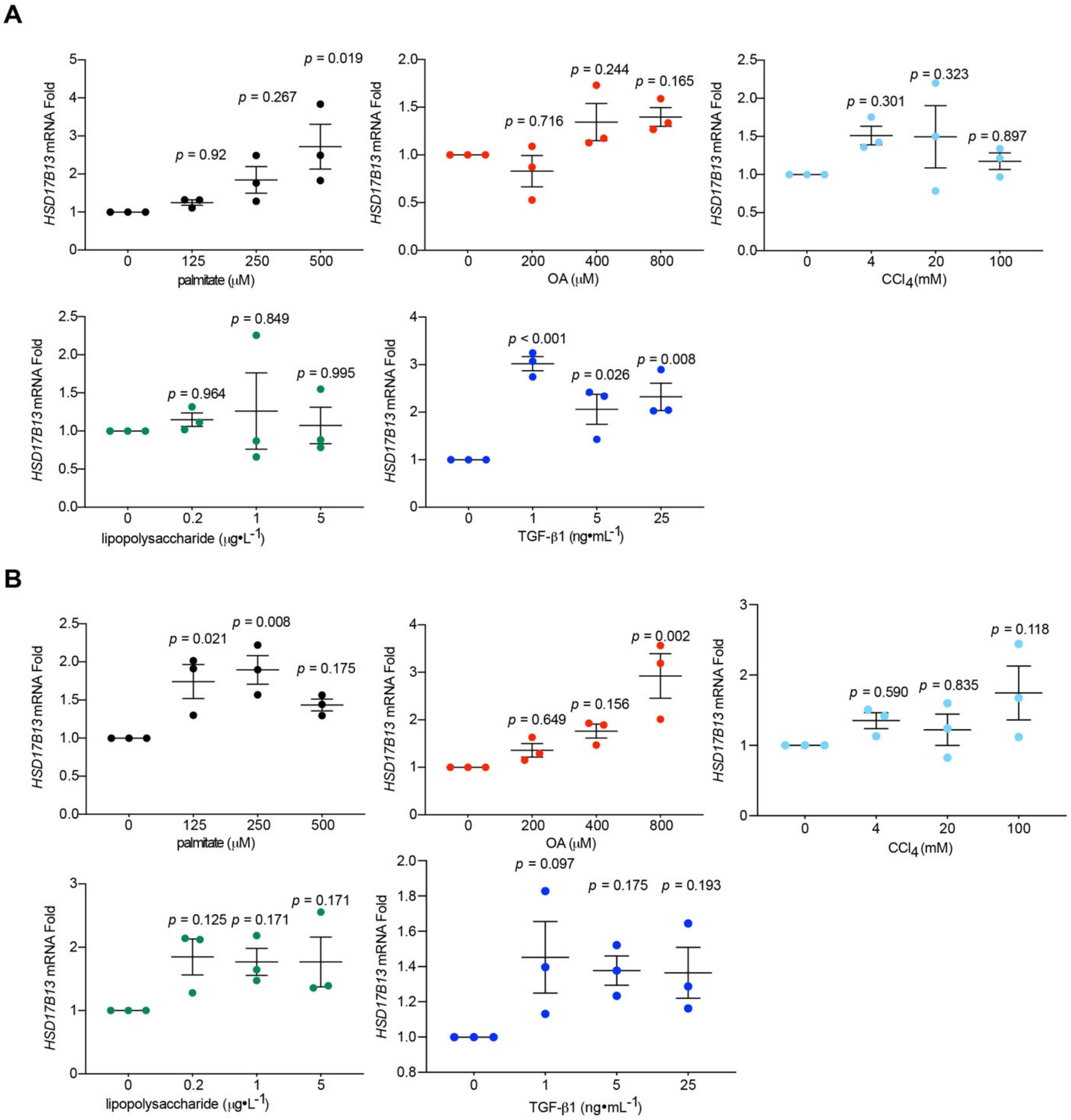

Supplementary Figure S2. HSD17B13 is increased in hepatocytes treated by etiological agents. Huh7 (A) and HepG2 (B) cells were treated by palmitate, OA, CCl_4, lipopolysaccharide, or TGF-β1 for 24 h, respectively. mRNAs were measured by qRT-PCR. Results are expressed as mean ± SEM. OA, oil acid; CCl_4, carbon tetrachloride; TGF-β1, transforming growth factor-beta 1. .

SUPPLEMENTARY TABLES

Supplementary Table S1. Patient information in DC-Liv00009 and H-Score for immunohistochemical analysis.

Patients number	Age	Sex	Organ	Pathology diagnosis	TNM	Grade	stage	HBV	H-Score
A1	45	Male	Liver	Normal	-	-	-	No info	13.08
A2	45	Male	Liver	Normal	-	-	-	No info	19.22
A3	23	Male	Liver	Normal	-	-	-	No info	21.57
A4	23	Male	Liver	Normal	-	-	-	No info	32.89
A5	27	Female	Liver	Normal	-	-	-	No info	21.13
A6	27	Female	Liver	Normal	-	-	-	No info	13.88
A7	42	Female	Liver	Normal	-	-	-	No info	31.01
A8	42	Female	Liver	Normal	-	-	-	No info	32.66
A9	36	Male	Liver	Normal	-	-	-	No info	50.9
A10	36	Male	Liver	Normal	-	-	-	No info	51.12
A11	21	Female	Liver	Normal	-	-	-	No info	42.33
A12	21	Female	Liver	Normal	-	-	-	No info	26.81
A13	43	Male	Liver	Normal	-	-	-	No info	39.3
A14	43	Male	Liver	Normal	-	-	-	No info	39.08
A15	50	Male	Liver	Normal	-	-	-	No info	23.1
A16	50	Male	Liver	Normal	-	-	-	No info	9.57
B1	16	Male	Liver	Normal	-	-	-	No info	72.02
B2	16	Male	Liver	Normal	-	-	-	No info	69.21
B3	45	Male	Liver	Normal	-	-	-	No info	84.96
B4	45	Male	Liver	Normal	-	-	-	No info	83.59
B5	38	Male	Liver	Normal	-	-	-	No info	67.14
B6	38	Male	Liver	Normal	-	-	-	No info	60.78
B7	40	Male	Liver	Normal	-	-	-	No info	61.05
B8	40	Male	Liver	Normal	-	-	-	No info	62.86
B9	35	Male	Liver	Normal	-	-	-	No info	61.18
B10	35	Male	Liver	Normal	-	-	-	No info	63.28
B11	35	Female	Liver	Normal	-	-	-	No info	79.21
B12	35	Female	Liver	Normal	-	-	-	No info	79.67
B13	23	Male	Liver	Normal	-	-	-	No info	71.05
B14	23	Male	Liver	Normal	-	-	-	No info	67.78
B15	18	Male	Liver	Normal	-	-	-	No info	73.97
B16	18	Male	Liver	Normal	-	-	-	No info	66.18
C1	58	Female	Liver	Inflammation	-	-	-	(-)	73.44
C2	58	Female	Liver	Inflammation	-	-	-	(-)	71.71
C3	60	Male	Liver	Inflammation	-	-	-	(+)	62.23
C4	60	Male	Liver	Inflammation	-	-	-	(+)	66.9
C5	48	Female	Liver	Inflammation	-	-	-	(+)	56.06
C6	48	Female	Liver	Inflammation	-	-	-	(+)	56.75

C7	72	Male	Liver	Inflammation	-	-	-	(-)	72.05
C8	72	Male	Liver	Inflammation	-	-	-	(-)	69.14
C9	52	Male	Liver	Inflammation<br>with steatosis	-	-	-	(-)	79.11
C10	52	Male	Liver	Inflammation	-	-	-	(-)	75.32
C11	33	Male	Liver	Inflammation	-	-	-	(+)	72.17
C12	33	Male	Liver	Inflammation	-	-	-	(+)	66.45
C13	42	Male	Liver	Inflammation<br>with steatosis	-	-	-	(-)	69.53
C14	42	Male	Liver	Inflammation<br>with steatosis	-	-	-	(-)	71.19
C15	56	Female	Liver	Inflammation	-	-	-	(+)	56.02
C16	56	Female	Liver	Inflammation	-	-	-	(+)	46.89
D1	69	Male	Liver	Inflammation	-	-	-	(+)	79.82
D2	69	Male	Liver	Inflammation	-	-	-	(+)	77.63
D3	55	Male	Liver	Inflammation<br>with steatosis	-	-	-	(+)	81.71
D4	55	Male	Liver	Inflammation	-	-	-	(+)	70.79
D5	49	Male	Liver	Inflammation	-	-	-	(-)	68.42
D6	49	Male	Liver	Inflammation<br>with steatosis	-	-	-	(-)	72.53
D7	56	Male	Liver	Inflammation	-	-	-	(+)	63.54
D8	56	Male	Liver	Inflammation	-	-	-	(+)	64.83
D9	68	Male	Liver	Inflammation	-	-	-	(+)	71.83
D10	68	Male	Liver	Inflammation	-	-	-	(+)	75.86
D11	56	Female	Liver	Inflammation	-	-	-	(+)	45.93
D12	56	Female	Liver	Inflammation<br>with steatosis	-	-	-	(+)	51.37
D13	48	Female	Liver	Inflammation	-	-	-	(+)	55.79
D14	48	Female	Liver	Inflammation	-	-	-	(+)	47.67
D15	45	Male	Liver	Inflammation<br>with steatosis	-	-	-	(+)	70.02
D16	45	Male	Liver	Inflammation	-	-	-	(+)	59.14
E1	62	Female	Liver	Inflammation<br>with steatosis	-	-	-	(+)	85.02
E2	62	Female	Liver	Inflammation<br>with steatosis	-	-	-	(+)	82.75
E3	55	Male	Liver	Inflammation	-	-	-	(-)	65.31
E4	55	Male	Liver	Inflammation	-	-	-	(-)	72.28

E5	40	Male	Liver	Inflammation	-	-	-	(-)	67.21
E6	40	Male	Liver	Inflammation	-	-	-	(-)	73.31
E7	55	Female	Liver	Inflammation	-	-	-	(-)	73.32
E8	55	Female	Liver	Inflammation	-	-	-	(-)	72.14
E9	57	Female	Liver	Inflammation	-	-	-	(-)	57.94
E10	57	Female	Liver	Inflammation	-	-	-	(-)	57.5
E11	35	Male	Liver	Inflammation	-	-	-	(+)	69.82
E12	35	Male	Liver	Inflammation	-	-	-	(+)	69.34
E13	67	Male	Liver	Inflammation	-	-	-	(-)	63.43
E14	67	Male	Liver	Inflammation	-	-	-	(-)	66.57
E15	52	Male	Liver	Inflammation	-	-	-	(+)	80.06
E16	52	Male	Liver	Inflammation	-	-	-	(+)	78.77
F1	60	Male	Liver	Cirrhosis	-	-	-	(+)	63.48
F2	60	Male	Liver	Cirrhosis	-	-	-	(+)	74.27
F3	52	Male	Liver	Cirrhosis	-	-	-	(+)	84.72
F4	52	Male	Liver	Cirrhosis	-	-	-	(+)	80
F5	43	Male	Liver	Cirrhosis	-	-	-	(+)	81.54
F6	43	Male	Liver	Cirrhosis	-	-	-	(+)	64.65
F7	50	Female	Liver	Cirrhosis	-	-	-	(-)	76.52
F8	50	Female	Liver	Cirrhosis	-	-	-	(-)	83.4
F9	25	Male	Liver	Cirrhosis	-	-	-	(-)	77.48
F10	25	Male	Liver	Cirrhosis	-	-	-	(-)	68.27
F11	52	Male	Liver	Cirrhosis	-	-	-	(+)	69.55
F12	52	Male	Liver	Cirrhosis	-	-	-	(+)	49.82
F13	40	Male	Liver	Cirrhosis	-	-	-	(-)	79.99
F14	40	Male	Liver	Cirrhosis	-	-	-	(-)	76.35
F15	48	Male	Liver	Cirrhosis	-	-	-	(+)	55.98
F16	48	Male	Liver	Cirrhosis	-	-	-	(+)	61.1
G1	47	Female	Liver	Cirrhosis	-	-	-	(-)	69.26
G2	47	Female	Liver	Cirrhosis	-	-	-	(-)	82.29
G3	36	Female	Liver	Cirrhosis	-	-	-	(-)	52.17
G4	36	Female	Liver	Cirrhosis	-	-	-	(-)	29.27
G5	35	Female	Liver	Cirrhosis	-	-	-	(+)	75.47
G6	35	Female	Liver	Cirrhosis	-	-	-	(+)	39.65
G7	61	Male	Liver	Cirrhosis	-	*	-	(-)	73.42
G8	61	Male	Liver	Cirrhosis	-	*	-	(-)	60.1
G9	46	Female	Liver	Cirrhosis	-	-	-	(+)	69.52
G10	46	Female	Liver	Cirrhosis	-	-	-	(+)	73.45
G11	67	Male	Liver	Cirrhosis	-	-	-	(+)	75.33
G12	67	Male	Liver	Cirrhosis	-	-	-	(+)	53.95
G13	42	Male	Liver	Cirrhosis	-	-	-	(+)	82.97
G14	42	Male	Liver	Cirrhosis	-	-	-	(+)	85.75
G15	66	Male	Liver	Cirrhosis	-	-	-	(+)	47.1
G16	66	Male	Liver	Cirrhosis	-	-	-	(+)	61.6

H1	67	Male	Liver	Cirrhosis	-	-	-	(+)	60.1
H2	67	Male	Liver	Cirrhosis	-	-	-	(+)	70.01
H3	49	Male	Liver	Cirrhosis	-	-	-	(-)	76.26
H4	49	Male	Liver	Cirrhosis	-	-	-	(-)	75.76
H5	45	Male	Liver	Cirrhosis	-	-	-	(-)	75.94
H6	45	Male	Liver	Cirrhosis	-	-	-	(-)	82.77
H7	38	Male	Liver	Cirrhosis	-	-	-	(+)	70.93
H8	38	Male	Liver	Cirrhosis	-	-	-	(+)	51.49
H9	60	Male	Liver	Cirrhosis	-	-	-	(+)	80.56
H10	60	Male	Liver	Cirrhosis	-	-	-	(+)	84.78
H11	46	Male	Liver	Cirrhosis	-	-	-	(-)	49.97
H12	46	Male	Liver	Cirrhosis	-	-	-	(-)	60.85
H13	54	Male	Liver	Cirrhosis	-	-	-	(-)	70.74
H14	54	Male	Liver	Cirrhosis	-	-	-	(-)	75.67
H15	66	Male	Liver	Cirrhosis	-	-	-	(-)	64.53
H16	66	Male	Liver	Cirrhosis	-	-	-	(-)	60.79
I1	37	Male	Liver	Cirrhosis	-	-	-	(+)	70.71
I2	37	Male	Liver	Cirrhosis	-	-	-	(+)	43.43
I3	46	Male	Liver	Cirrhosis	-	-	-	(-)	72.02
I4	46	Male	Liver	Cirrhosis	-	-	-	(-)	61.91
I5	43	Female	Liver	Cirrhosis	-	-	-	(-)	76.89
I6	43	Female	Liver	Cirrhosis	-	-	-	(-)	82.7
I7	70	Male	Liver	Cirrhosis	-	-	-	(+)	74.95
I8	70	Male	Liver	Cirrhosis	-	-	-	(+)	85.44
I9	28	Male	Liver	Malignant	T3N0M0	-	IIIA	(-)	73.08
I10	28	Male	Liver	Malignant	T3N0M0	2	IIIA	(-)	43.97
I11	49	Male	Liver	Malignant	T2N0M0	2	II	(-)	76.4
I12	49	Male	Liver	Malignant	T2N0M0	2	II	(-)	79.28
I13	53	Male	Liver	Malignant	T3N0M0	2	IIIA	(+)	61.79
I14	53	Male	Liver	Malignant	T3N0M0	2	IIIA	(+)	41.41
I15	55	Male	Liver	Malignant	T3N0M0	3	IIIA	(+)	29.68
I16	55	Male	Liver	Malignant	T3N0M0	3	IIIA	(+)	31.37
J1	41	Male	Liver	Malignant	T2N0M0	1--2	II	(+)	53.64
J2	41	Male	Liver	Malignant	T2N0M0	1--2	II	(+)	51.61
J3	52	Male	Liver	Malignant	T3N1M0	2	IVA	(+)	0.32
J4	52	Male	Liver	Malignant	T3N1M0	2	IVA	(+)	0.16
J5	60	Male	Liver	Malignant	T3N0M0	2	IIIA	(+)	3.34
J6	60	Male	Liver	Malignant	T3N0M0	2	IIIA	(+)	2.73
J7	50	Female	Liver	Malignant	T2N0M0	3	II	(-)	6.84
J8	50	Female	Liver	Malignant	T2N0M0	3	II	(-)	8.17
J9	48	Male	Liver	Malignant	T2N0M0	2	II	(+)	57.54
J10	48	Male	Liver	Malignant	T2N0M0	2	II	(+)	59.25
J11	55	Male	Liver	Malignant	T3N0M0	3	IVA	(+)	19.05
J12	55	Male	Liver	Malignant	T3N0M0	3	IVA	(+)	6.37

J13	35	Female	Liver	Malignant	T2N0M0	3	II	(+)	7.81
J14	35	Female	Liver	Malignant	T2N0M0	3	II	(+)	3.64
J15	50	Male	Liver	Malignant	T3N0M0	3	IIIA	(+)	45.63
J16	50	Male	Liver	Malignant	T3N0M0	3	IIIA	(+)	35.21
K1	46	Male	Liver	Malignant	T2N0M0	2	II	(-)	34.38
K2	46	Male	Liver	Malignant	T2N0M0	2	II	(-)	6.62
K3	66	Male	Liver	Malignant	T3N0M0	3	IIIA	(-)	20.92
K4	66	Male	Liver	Malignant	T3N0M0	3	IIIA	(-)	20.33
K5	56	Female	Liver	Malignant	T3N0M0	*	IIIA	(+)	20.6
K6	56	Female	Liver	Malignant	T3N0M0	*	IIIA	(+)	15.36
K7	38	Male	Liver	Malignant	T3N0M0	3	IIIA	(+)	52.82
K8	38	Male	Liver	Malignant	T3N0M0	3	IIIA	(+)	47.21
K9	54	Male	Liver	Malignant	T3N0M0	3	IIIA	(-)	4.42
K10	54	Male	Liver	Malignant	T3N0M0	3	IIIA	(-)	5.12
K11	62	Male	Liver	Malignant	T3N0M0	3	IIIA	(+)	67.25
K12	62	Male	Liver	Malignant	T3N0M0	3	IIIA	(+)	70.82
K13	58	Male	Liver	Malignant	T3N0M0	3	IIIA	(+)	11.11
K14	58	Male	Liver	Malignant	T3N0M0	3	IIIA	(+)	8.01
K15	62	Male	Liver	Malignant	T2N0M0	3	II	(+)	16.07
K16	62	Male	Liver	Malignant	T2N0M0	3	II	(+)	5.33
L1	67	Male	Liver	Malignant	T2N0M0	3	II	(+)	12.7
L2	67	Male	Liver	Malignant	T2N0M0	3	II	(+)	14.73
L3	62	Male	Liver	Malignant	T3N0M0	3	IIIA	(+)	58.06
L4	62	Male	Liver	Malignant	T3N0M0	3	IIIA	(+)	54.38
L5	59	Male	Liver	Malignant	T3N0M0	3	IIIA	(+)	79.11
L6	59	Male	Liver	Malignant	T3N0M0	3	IIIA	(+)	76.33
L7	45	Male	Liver	Malignant	T3N0M0	2	IIIA	(+)	19.5
L8	45	Male	Liver	Malignant	T3N0M0	2	IIIA	(+)	21.01
L9	32	Male	Liver	Malignant	T4N0M0	3	IIIB	(+)	19.14
L10	32	Male	Liver	Malignant	T4N0M0	3	IIIB	(+)	26.26
L11	64	Male	Liver	Malignant	T3N0M0	3	IVA	(+)	3.27
L12	64	Male	Liver	Malignant	T3N0M0	3	IVA	(+)	2.83
L13	52	Male	Liver	Malignant	T3N0M0	3	IIIA	(+)	31.88
L14	52	Male	Liver	Malignant	T3N0M0	3	IIIA	(+)	27.22
L15	48	Female	Liver	Malignant	T2N0M0	2	II	(+)	1.21
L16	48	Female	Liver	Malignant	T2N0M0	2	II	(+)	0.6

Supplementary Table S2 . Antibodies for Western blot and immunohistochemical staining analyses.

Antibody	Source	Cat No.
COL1A1	Santa Cruz	sc-293182
TIMP1	Santa Cruz	sc-365905
β-Actin	Cell Signaling Technology	3700S
SCD1	Cell Signaling Technology	2438S
ACC1	HUABIO	ET1609-77
PLIN2	HUABIO	ET1704-17
HSD17B13	NOVUS	NBP3-04837
IBA-1	Servicebio	GB12105
CD31	Servicebio	GB113151
CD11b	Servicebio	GB11058
CD36	Abcam	ab133625
HCV core	Abcam	ab2740
HSD17B13	Abcam	ab122036
α-SMA	Abcam	ab7817
Albumin	Abcam	ab106582
6X-his tag	Invitrogen	MA121315
HSD17B13	Invitrogen	PA5-56063
HSD17B13	Invitrogen	PA5-25633
HSD17B13	Invitrogen	PA5-109834

Supplementary Table S3. Primers for plasmid construction.

Gene	Primer Sequence (5'-3')	
HSD17B13	Forward	TACCGAGCTCGGATCCATGAACATCATCCTAGAAATCC
	Reverse	AACGGGCCCTCTAGACTCGAGTCATTTCATTTTGATTTTGT
HSD17B13-His	Reverse	AACGGGCCCTCTAGACTCGAG-<br>TCAATGGTGATGGTGATGATGTTTCATTTTGATTTTGT
HSD17B13 iso-D	Forward	CCTGAACGCGCCTCAGCGATTTTAAATGGTATGCAG
	Reverse	TGAGGCGCGTTCAGGAAGAAACTTCTGTAGTCTCAG
PLIN2	Forward	CGGGGTACCATGGCATCCGTTGCAGTTGATCC
	Reverse	CCCTCGAGTCAATGAGTTTTATGCTCAGAT

Supplementary Table S4. Primers for qRT-PCR.

Gene		Sequence (5'-3')
Human *GAPDH*	Forward	CGGAGTCAACGGATTTGGTCGTAT
	Reverse	AGCCTTCTCCATGGTGGTGAAGAC
Human *HSD17B13*	Forward	ATCACAAAAGCACTTCTTCCATC
	Reverse	AGACCTCTGTGAAAGCCAAC
Human *HSD17B4*	Forward	TTGGGCCGAGCCTATGC
	Reverse	CCCCTCCCAAATCATTCACA
Human *HSD17B7*	Forward	TTGACACCATATAATGGAACAGAAG
	Reverse	TGATCAGAGGATTGAGAGATTCAG
Human *HSD17B8*	Forward	ACATCAGTAGCATCGTAGG
	Reverse	GGAGGACAGAGTTACAGC
Human *HSD17B10*	Forward	GGCATGACACTGCCCATTG
	Reverse	GGTCACCCAGTCGGCTAGG
Human *HSD17B11*	Forward	TGCAATGACGAAGAATAACC
	Reverse	TTGTAAGGCAGCCAGTTC
Human *HSD17B12*	Forward	GCCAACTTTGGATAAGCCCTCTC
	Reverse	AGGCAGGTTTGAGATTATCGAGC
Human *α-SMA*	Forward	GAGTTACGAGTTGCCTGATGG
	Reverse	GATGCTGTTGTAGGTGGTTTCA
Human *COL1A1*	Forward	TCTGGCGCTCCCATGGCTCT
	Reverse	GCCCTGCGGCACAAGGGATT
Human *TIMP1*	Forward	TGTTGTTGCTGTGGCTGATAGC
	Reverse	TCTGGTGTCCCCACGAACTT
HCV	Forward	CGGGAGAGCCATAGTGGTCTGCG
	Reverse	CTCGCAAGCACCCTATCAGGCAGTA
	Probe	FAM-5′-AGGCCTTGTGGTACTGCCT-3′-TAMRA
GAPDH [a]	Forward	CGGAGTCAACGGATTTGGTCGTAT
	Reverse	AGCCTTCTCCATGGTGGTGAAGAC
	Probe	FAM-5′-CCGTCAAGGCTGAGAACGG-3′-TAMRA
Human *IL-6*	Forward	GAGTAGTGAGGAACAAGCCAGA
	Reverse	AAGCTGCGCAGAATGAGATGA
Human *CXCL3*	Forward	CCAAACCGAAGTCATAGCCAC
	Reverse	TGCTCCCCTTGTTCAGTATCT
Human *TGF-β1*	Forward	CCGACTACTACGCCAAGGAG
	Reverse	TGAGGTATCGCCAGGAATTG
Mouse *Hsd17b13*	Forward	TTGCAAAGCAGAAAAGCAGA
	Reverse	GGCGTTGTTTACCACGATCT

[a]. internal reference for HCV quantification.
